# AF4-to-SAXS: expanded characterization of nanoparticles and proteins at the P12 BioSAXS beamline

**DOI:** 10.1107/S1600577525003959

**Published:** 2025-06-12

**Authors:** Stefano Da Vela, Kim Bartels, Daniel Franke, Dymtro Soloviov, Tobias Gräwert, Dmitry Molodenskiy, Bastian Kolb, Christoph Wilhelmy, Roland Drexel, Florian Meier, Heinrich Haas, Peter Langguth, Melissa A. Graewert

**Affiliations:** a European Molecular Biology Laboratory Hamburg Unit Hamburg Germany; b Department of Biopharmaceutics and Pharmaceutical Technology Johannes Gutenberg University Mainz Germany; c BIOSAXS GmbH, Hamburg, Germany; d Postnova Analytics GmbH Landsberg am Lech Germany; NSRRC, Taiwan

**Keywords:** AF4, biological SAXS, P12 beamline, proteins, nanoparticles, full automation, operator-friendly guidelines

## Abstract

Biological small-angle X-ray scattering (SAXS) is a versatile and powerful technique for investigating the structural and biophysical properties of biologically and pharmaceutically relevant macromolecules and nanoparticles. SAXS offers detailed insights into macromolecular composition, size, shape and internal structure, while addressing key aspects such as oligomeric state, stability, molecular interactions, and conformational flexibility. Recently, asymmetrical-flow field-flow fractionation (AF4) was successfully coupled to SAXS, enabling online size-based fractionation and analysis of polydisperse samples. This approach allows precise, size-dependent characterization, offering significant advancements in the study of polydisperse systems. We have integrated an AF4 device at the P12 beamline at the European Molecular Biology Laboratory and implemented technical adaptations allowing full automation to make the system suitable for routine user access. We provide streamlined workflows and troubleshooting resources for both novice and advanced SAXS users thereby equipping them with clear guidance on performing AF4–SAXS measurements. The general principles of our set-up are easily adaptable to other beamlines which have integrated (or are planning to integrate) a similar system.

## Abstract 2

By coupling asymmetrical-flow field-flow fractionation to small-angle X-ray scattering (AF4–SAXS), we enable precise, size-resolved analysis of polydisperse samples. Our automated AF4–SAXS system at the EMBL P12 beamline streamlines workflows, making advanced characterization accessible to both novice and experienced users, with principles adaptable to other facilities.

## 1. Introduction

The colloidal domain (Fennell Evans & Wennerström, 1999 ▸) has become an integral part of material science and bio­medical research, with nanoparticles, finding applications in diverse fields, including drug delivery, environmental monitoring, and consumer products as prominent representatives. While a variety of different materials, organic and inorganic, metallic, fully synthetic as well as bio-based, can be applied for nanoparticle formation, they all share some common structural–functional coherencies. Understanding structural aspects of these systems at different length scales remains a challenge (Clogston *et al.*, 2020 ▸). Small-angle X-ray scattering (SAXS) has proven to be a powerful tool for characterizing size, shape, interactions, and conformational flexibility in solution, providing insights into biological and synthetic macromolecules (Bernadó *et al.*, 2018 ▸; Brosey & Tainer, 2019 ▸; DaVela & Svergun, 2020 ▸; Uebbing *et al.*, 2020 ▸; Urimi *et al.*, 2022 ▸; Trewhella, 2022 ▸; Sonje *et al.*, 2023 ▸; Mohammed *et al.*, 2024 ▸). SAXS experiments benefit from precise sample preparation and separation techniques to resolve heterogeneous systems and maximize the extraction of reliable information from the systems studied (Jeffries *et al.*, 2016 ▸; Trewhella *et al.*, 2023 ▸).

Nanoparticle systems are very often polydisperse by their nature, which hampers the unequivocal interpretation of data from ensemble measurements of these systems. Therefore, attempts have been made to apply separation techniques prior to the SAXS measurements, for example, filtration or centrifugation to reduce large aggregates, or size-separating techniques such as size exclusion chromatography (SEC; see reviews by Pérez & Vachette, 2017 ▸; Pérez *et al.*, 2022 ▸). Because of potential column interactions of the analyte, and other disadvantages such as the dilution effect (Grinias *et al.*, 2015 ▸), there is a need for coupling of alternative (size) separation techniques to SAXS for the systems where SEC is not effective.

Asymmetrical-flow field-flow fractionation (AF4) is a less invasive, well established method to separate particulates and proteins as a function of size (Giddings, 1993 ▸; Schimpf *et al.*, 2000 ▸). Over the last decades, AF4 has been applied and established in various application areas, like polymer and biopolymer sciences (Tank & Antonietti, 1996 ▸; Wittgren & Wahlund, 1997 ▸; Podzimek *et al.*, 2009 ▸), virus and virus-like particle characterization (Litzen & Wahlund, 1989 ▸; Chuan *et al.*, 2008 ▸; Eskelin *et al.*, 2016 ▸), nucleic acids (Ma *et al.*, 2010 ▸; Ashby *et al.*, 2014 ▸) and more advanced materials in nano­medical applications (Wagner *et al.*, 2014 ▸; Parot *et al.*, 2020 ▸). AF4 can be coupled to SAXS, offering the ability to separate components by size in a gentle, matrix-free environment, and has recently been demonstrated by us and others (Thünemann *et al.*, 2009 ▸; Bolinsson *et al.*, 2023 ▸; Graewert *et al.*, 2023 ▸; Börjesdotter *et al.*, 2025 ▸). So far, the adaption of AF4–SAXS workflows for investigating a larger scope of nanoparticulate products has been hampered by the fact that AF4 technology is unfamiliar to many SAXS users.

To overcome this limitation, we have integrated AF4 into the beamline operation in a fully automated manner, significantly streamlining the workflow and reducing the entry barrier for new users. This integration ensures that even those with limited experience in AF4 can achieve reliable and reproducible results. Multi-angle laser light scattering (MALS) data can be collected in parallel to obtain scattering data at a lower *q*-range than is usually the case with SAXS. This enables the extraction of critical information, including independent molecular weight (MW) estimates for proteins and, for larger nanoparticles (≥10 nm), the determination of the radius of gyration (*R*_g_).

In this study, we demonstrate the versatility and capability of this automated AF4–SAXS platform by investigating a variety of systems covering different length scales, including the separation of oligomeric states of individual proteins and nanoparticles of varying sizes, using nanometre-size polystyrene (PS) beads for proof-of-concept measurements.

However, this approach is intentionally simplified to provide an accessible entry point for SAXS users who are new to AF4. The concepts demonstrated, using standard samples that are commonly employed in both the AF4 and SAXS communities, are intended to offer a foundational understanding. These concepts may not be universally applicable in all scenarios. Key challenges include sample–membrane interactions, recovery efficiency, carrier liquid selection, and dilution effects—each of which requires careful optimization. For polydisperse nanoparticles, delicate protein assemblies, or complex mixtures, a more thorough optimization of AF4 parameters is essential. Users should adjust their expectations accordingly and be prepared for iterative testing to achieve optimal separation before collecting SAXS data of sufficient quality.

The theory and challenges of establishing AF4 separation protocols have been extensively reviewed elsewhere (Giddings *et al.*, 1987 ▸; Schimpf *et al.*, 2000 ▸; Baalousha *et al.*, 2011 ▸; Wahlund, 2013 ▸; Gigault *et al.*, 2014 ▸; Eskelin *et al.*, 2019 ▸; Quattrini *et al.*, 2021 ▸). Here, we highlight and visualize key concepts by presenting the separation of bovine serum albumin (BSA) as a representative example. The data presented from the optimization of off-line measurements aim to assist novice users in refining their AF4 separation method. This guidance emphasizes critical parameters and procedural adjustments that can be fine-tuned to achieve optimal outcomes, including (but not limited to) variations in channel geometry, flow rates, and elution times as well as sample loading conditions. Finally, we draw parallels with and differences from the well established and routinely used size-exclusion chromatography coupled SAXS (SEC–SAXS), particularly in terms of sample dilution and its impact on SAXS data quality.

## 2. Results and discussion

### 2.1. Key AF4 principles to consider when combining AF4 with SAXS

**Figure 1:**
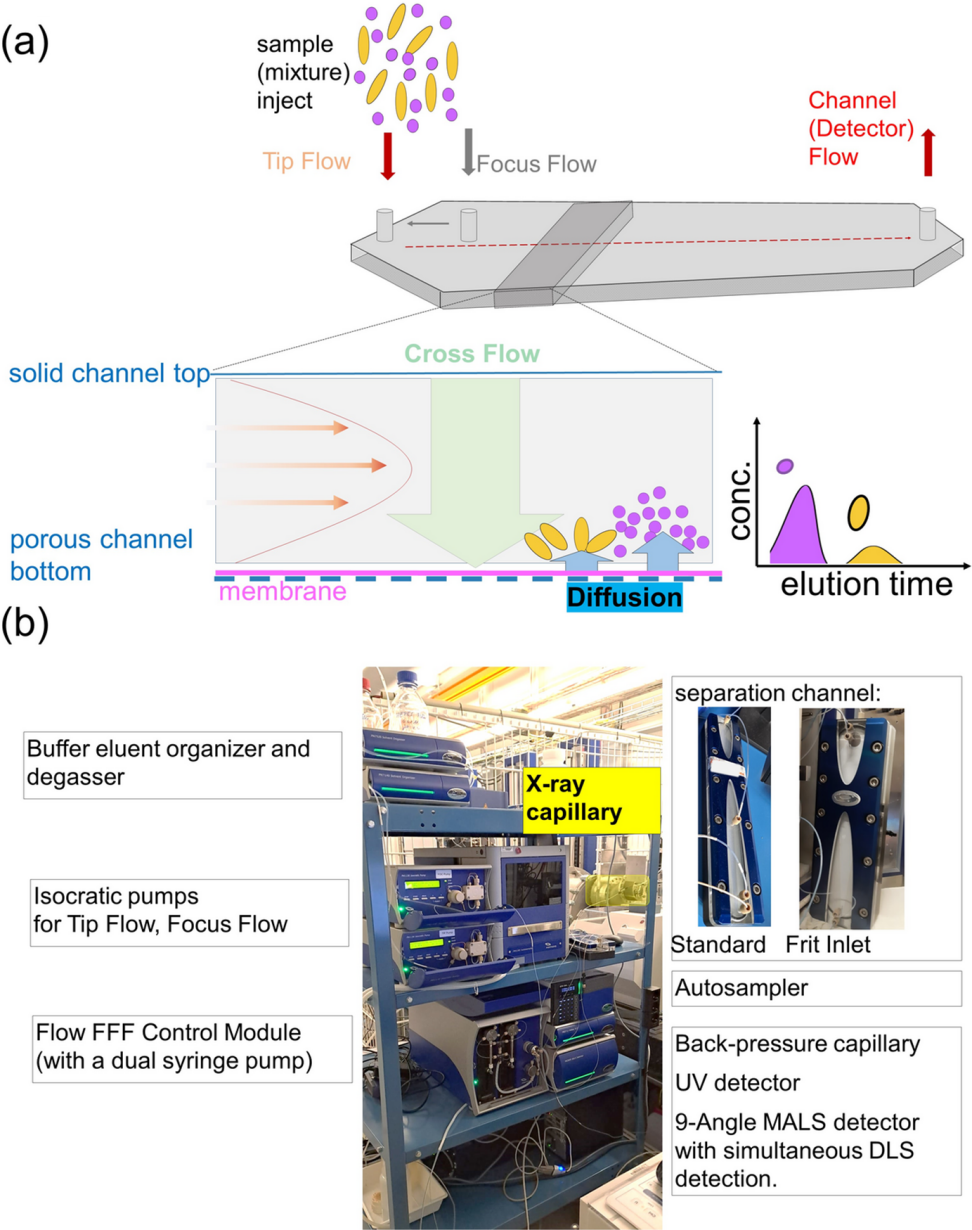
Overview and hardware integration. (*a*) Schematic overview of the separation process as achieved through AF4, illustrating the flow dynamics and fractionation principles. (*b*) AF4–SAXS instrumentation integrated into the P12 beamline, featuring a trolley-mounted setup for seamless transitions between offline and online configurations. The inset highlights the various channels available for separation, accommodating different sample requirements. Further details are provided in the main text.

AF4 is a specialized variant of the broader field-flow fractionation (FFF) technique [as described by Giddings in a 1966 review (Giddings, 1966 ▸)], which separates components based on their diffusion coefficients in a laminar flow field. The AF4 concept was first published by Wahlund & Giddings (1987 ▸) and further optimized in the following years (*e.g.* Wahlund & Litzén, 1989 ▸). The basic principle behind FFF and in particular AF4 is that a laminar flow is established in an open channel, where the fluid moves smoothly in parallel layers, with slower velocities near the channel walls and faster velocities at the centre [Fig. 1 ▸(*a*), orange arrows].

In the case of AF4, the separation is achieved by the addition of a perpendicular flow force, which refers to the application of Cross Flow [Fig. 1 ▸(*a*), green arrow] perpendicular to the main flow. This Cross Flow directs the particles toward a semi-permeable membrane (accumulation wall) with a specific cut-off, allowing smaller particles—and, importantly, larger volumes of the liquid medium—to pass through, while larger particles are retained within the channel (Wahlund & Giddings, 1987 ▸). Typically, the membrane cut-off is 10 kDa, but smaller and larger meshes are available (between 300 Da and 150 kDa). Larger particles, with lower diffusion rates [Fig. 1 ▸(*a*), blue arrows], remain closer to the membrane (yellow beads), while smaller particles, which show higher diffusivity (purple spheres), distribute further from the membrane, toward the channel centre. This size-dependent segregation facilitates the separation of sample components. Thus, smaller particles elute first, followed by progressively larger ones. By adjusting the Cross Flow rate, the separation can be fine-tuned to achieve high resolution, even for complex and polydisperse samples.

The described elution mode is called Brownian mode. When the particle size increases above a certain size limit, which is approximately in the range of 1 or 2 µm, the Brownian motion becomes negligible. Particles are held by the separation force on the accumulation wall and the fractionation process is inverted, which means that larger particles elute before smaller ones. This mode is called steric/hyperlayer mode (Schimpf *et al.*, 2000 ▸; Giddings *et al.*, 1987 ▸).

At P12, we prioritize the analysis of samples below 0.5 µm, in accordance with detection limits at the beamline, and recommend the removal of larger particles before the experiment. This can be achieved through filtration (0.45 µm) or centrifugation, which allows separation to occur predominantly in Brownian mode, preventing steric hyperlayer effects.

During the injection phase, two isocratic pumps are used to generate an Input Flow, referred to as Tip Flow (red arrow) and Focus Flow (grey arrow). The Tip Flow is connected to the automatic sample loader and used for sample injection. The Focus Flow realizes the focusing and relaxation of the sample constituents near the accumulation wall, which reduces band broadening to a minimum, allowing for improved signals due to the higher number density of particles ultimately in the X-ray beam [Fig. S1(*a*) of the supporting information]. The flow which eventually reaches the X-ray flow-through capillary is referred to as Detector Flow (FL_Det_), and is the difference between the combined Input Flow (from Tip Flow, FL_Tip_, and Focus _Flow_, FL_Foc_) and the Cross Flow, FL_Cross_,
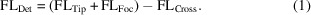
In summary, the overall separation technique of AF4 relies on the interplay of laminar channel flow, Cross Flow, and diffusion to achieve size-based separation. This approach ensures a precise and reproducible method for fractionating a wide range of particle sizes, from small molecules to larger macromolecules and nanoparticles, without the need of a stationary phase interacting with the sample. An overview of the influence on the separation resolution by adapting these different flow parameters is given below (see Section 2.6).

The quality of the separation can then be assessed by using the Detector Flow (red arrow) to push particles through a range of detectors. These can include detectors such as UV, refractive index (RI), multi-angle light scattering (MALS), or, as described here, SAXS [Fig. 1 ▸(*a*)].

It should be noted that solvent exchange in an AF4 channel is not directly analagous to SEC; in SEC–SAXS, solvent exchange is typically performed by the column in a way that allows for accurate buffer subtraction. However, with AF4 the completeness of the solvent exchange depends on the permeability of the membrane, which is more or less related to its MWCO: lower-MWCO membranes result in slower or less complete solvent exchange. As highly accurate buffer subtraction is critical to SAXS data analysis, it is a useful experimental recommendation to have samples already in exactly matched buffer as they would be for batch SAXS. In some cases, another recommendation for better buffer matching would be to select higher MWCO membranes (*i.e.* ≥30 kDa+) when possible.

### 2.2. Hardware integration at the bioSAXS beamline P12

Two major commercial systems dominate the AF4 market, both of which have been successfully integrated into SAXS beamlines setups based on experimental demonstrations: (i) the Wyatt eclipse system at the CoSAXS beamline at MAX IV in Lund, Sweden (Bolinsson *et al.*, 2023 ▸; Börjesdotter *et al.*, 2025 ▸) and reported by other beamlines (such as SIBYLS at ALS and BioCAT at APS in the USA); (ii) the POSTNOVA AF2000 system, which is the system we have integrated at P12 and describe in this study [Fig. 1 ▸(*b*)]. An identical system was used for the experiments described by Graewert *et al.* (2023 ▸).

Key elements of the AF4 system include:

(*a*) AF2000 module: the Flow FFF Control Module with a dual syringe pump to generate the reproducible and stable Cross Flow while minimizing any pulsation.

(*b*) Two isocratic pumps to generate both Tip Flow and Focus Flow.

(*c*) An eluent organizer and eluent degasser.

(*d*) For precise loading and importantly remote operation, an automatic sample loader is in place which uses a pressure-assisted sample aspiration. It is temperature-controlled, allows for different washing programs, and offers a sample shaking mode intended to prevent sedimentation of larger particles.

(*e*) The set-up is controlled with a PC and the commercially licenced *NovaFFF* software (Postnova Analytics GmbH, Landsberg am Lech, Germany).

(*f*) Currently, two different separation channels are available at P12: a standard channel with the possibility of applying a focus stream (as described above) as well as a semi-preparative channel as a frit-inlet channel (which minimizes contact with the accumulation wall and reduces shear forces by applying a Frit Flow via a porous frit-inlet to realize hydro­dynamic relaxation (Fig. S1).

(*g*) For controlled separation a backpressure capillary is directly coupled to the outlet of the channel.

(*h*) A UV detector comprising two channels with selectable wavelength (190–700 nm), 10 mm path length and 12 µL cell volume is placed inline for detecting eluted sample components through absorption measurements.

(*i*) A nine-angle multi-angle light scattering (MALS) detector comprising a vertical flow cell.

(*j*) Online dynamic light scattering (DLS) detection through an optical fibre connection to the MALS detector.

(*k*) SAXS capillary – this device is integrated in the automated sample loader through a valve which allows seamlessly switching of the cell to ‘flow-through mode’ so that the AF4 channel eluent can pass through the SAXS capillary.

To facilitate full integration at the beamline, several components have been integrated to enhance the functionality and facilitate operation of the AF4 system:

(*l*) Trolley for transportation: the system is mounted on a trolley, enabling easy transport between off-line and on-line setups. The off-line development of separation techniques ensures that methods are fully optimized before coupling to SAXS.

(*m*) An additional UPS (uninterruptible power supply) has been integrated into the system to ensure consistent flow rates while switching between off-line and on-line operation. This addition optimizes beam time by providing flexibility and adapting to the accessibility of the hutch, ensuring un­interrupted operation during transitions and maintaining system stability throughout experiments.

(*n*) 3D-printed sample holders: custom-designed 3D-printed holders were created for the efficient loading of biological samples. These holders ensure secure placement and easy handling, reducing the risk of contamination or spillage.

### 2.3. Software integration for fully automated data collection

**Figure 2:**
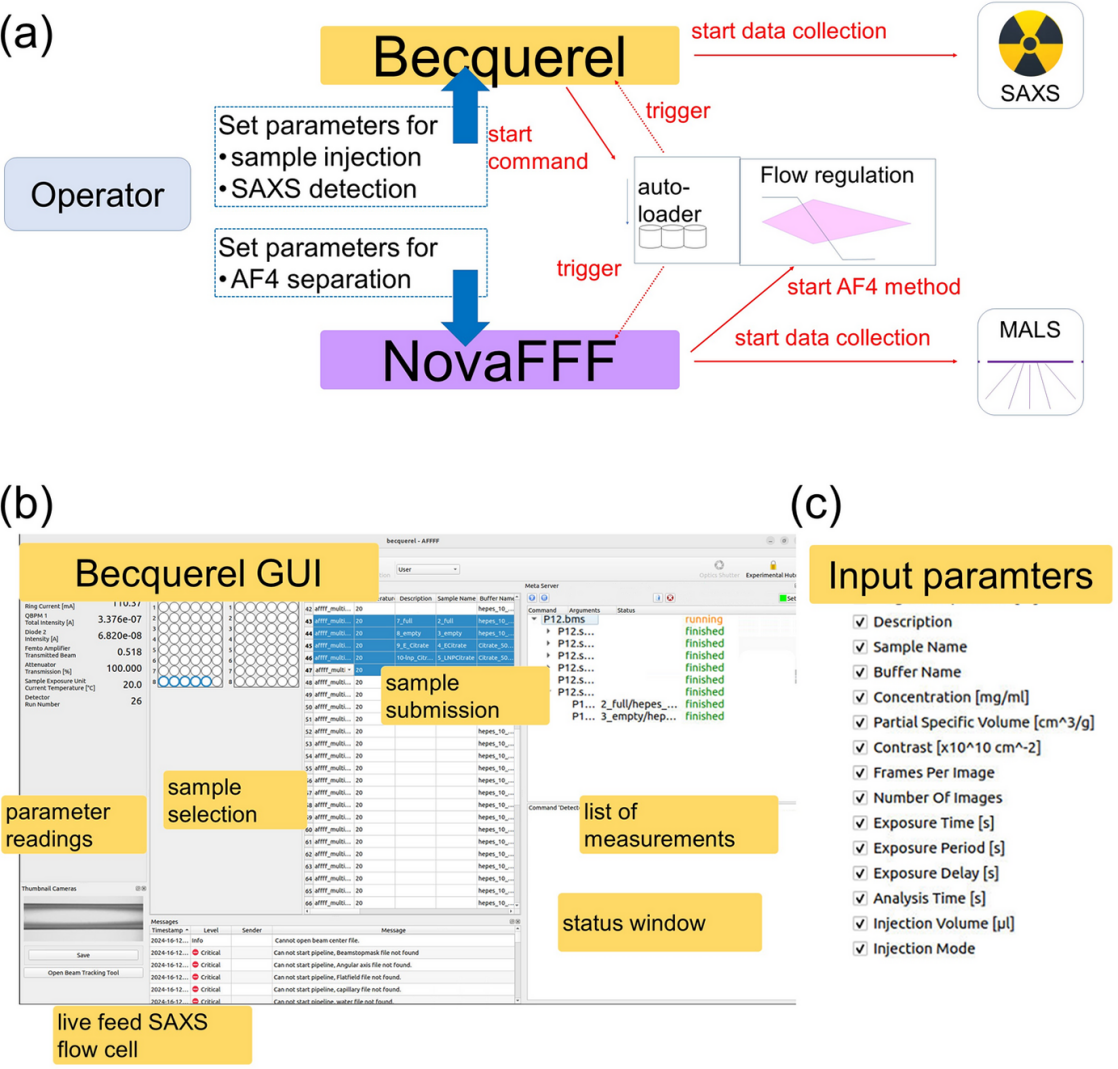
Workflow and software integration. (*a*) Schematic overview depicting the integration of the AF4 system and *NovaFFF* software into the SAXS beamline control system, *BECQUEREL*. The workflow illustrates the information flow from the beamline operator through input fields to data collection. The beamline meta server sends a command to the automated sample loader for sample injection. Once the injection is confirmed, SAXS data frames are recorded while *NovaFFF* regulates the flow according to the programmed separation protocol and simultaneously records UV and MALS data. (*b*) Screenshot of the *BECQUEREL* graphical user interface (GUI) in AF4 mode. (*c*) List of selectable input parameters provided in *BECQUEREL* for the beamline operator to configure experiments.

To streamline operations and enhance usability, the AF4 system was fully integrated into the *BECQUEREL* control software used at the beamline (Franke *et al.*, 2012 ▸; Hajizadeh, Franke & Svergun, 2018 ▸). This integration enables synchronized operation of AF4 flows and UV/MALS detection with SAXS data acquisition, as shown in the communication workflow in Fig. 2 ▸(*a*).

Operators place the sample(s) into the automated sample loader and set the interlock system to prepare for usage of the X-rays. In the *BECQUEREL* graphical user interface (GUI) the data collection parameters are set, which includes input on sample details [*e.g.* name, concentration, and loading volume, as illustrated in Figs. 2 ▸(*b*) and 2(*c*)], along with SAXS collection settings such as exposure time (typically 1 s, depending on attenuation, Detector Flow rate, and sample sensitivity to radiation damage in the carrier buffer) and the number of frames, which depend on the required elution time for sufficient separation. After submission through the *BECQUEREL* GUI, the Beamline Meta Server (BMS) coordinates the automated data collection. This includes, for example, a system check (state of the synchrotron ring current, vacuum readings, *etc*.) and detector preparation at the beginning of the SAXS experiment. This ensures that all beamline components are idle and ready and that required permissions, such as writing rights for the images in the selected paths, are given.

For triggering the data collection, a routine has been added to BMS which includes:

(*a*) Sample injection: initiates injection via the POSTNOVA automatic sample loader.

(*b*) A signal is received by the *NOVAFFF* software, which triggers:

 (i) The start of the elution sequence which requires regulation of flow rates (Tip, Focus, and Cross Flows)

 (ii) The start of complementary data (*e.g.* MALS and UV data) collection.

(*c*) After the BMS registers the successful injection, recording of the SAXS data frames is initiated at the detector.

The duration of the experiment is determined by the overall number of frames as well as the AF4 separation method. At the end of the run, a rinse step is in place to make sure all particles are removed from the channel. In parallel, operators are encouraged to enable the automatic SAXS capillary rinsing program between sample injections to remove potential fouling caused by prolonged X-ray exposure which may manifest itself as particle build-up on the SAXS capillary wall.

The individual runs may be submitted as a sequence. For this, a table including all run parameters is prepared in the *NovaFFF* software, and the corresponding sample names, vial position, and loading volume are entered into the *BECQUEREL* software. For this purpose, it is important that the duration of the scheduled SAXS data collection is slightly longer than the actual AF4 separation program so that the AF4 system is idle before the subsequent sample is injected.

After the completion of an individual run, the processing of the SAXS data is initialized with the SAXS pipeline (*SASFLOW*; Franke *et al.*, 2012 ▸; Manalastas-Cantos *et al.*, 2021 ▸) which ultimately converts 2D scattering patterns into 1D curves performing standard corrections, radial averaging, and normalizations. The program *CHROMIXS* can then be used to visualize the outcome of the SAXS experiment (Panjkovich & Svergun, 2018 ▸). Here, an elution profile (SAXS fractogram) is generated by integrating selected *q* ranges for each individual 1D curve and plotting these values as integrated SAXS intensity versus frames number. The advantages of *CHROMIXS* are described elsewhere (Manalastas-Cantos *et al.*, 2021 ▸; Franke *et al.* 2025 ▸) but, briefly, it allows operators to select and choose frames corresponding to the scattering of particles of interest. In the first step, frames representing the sample are selected, followed by the selection of buffer frames. These are then processed to provide a final scattering curve used for analysis of the sample. For more sophisticated analysis, scripts are in place to analyse individual fractions to retrieve sized-dependent parameters on the overall fold or internal structure of the sample at different elution points.

In summary, by integrating these hardware and software components, the AF4–SAXS platform delivers a seamless, user-friendly experience while maintaining the adaptability required for diverse samples and experimental setups.

Next, the platform’s capabilities are demonstrated through the separation and analysis of standard samples.

### 2.4. Separation across length scales

In general, the strategy for AF4 separation varies significantly between smaller particles such as proteins and nucleic acids (typically smaller than 20 nm) and larger nanoparticles.

#### 2.4.1. Constant Cross Flow

**Figure 3:**
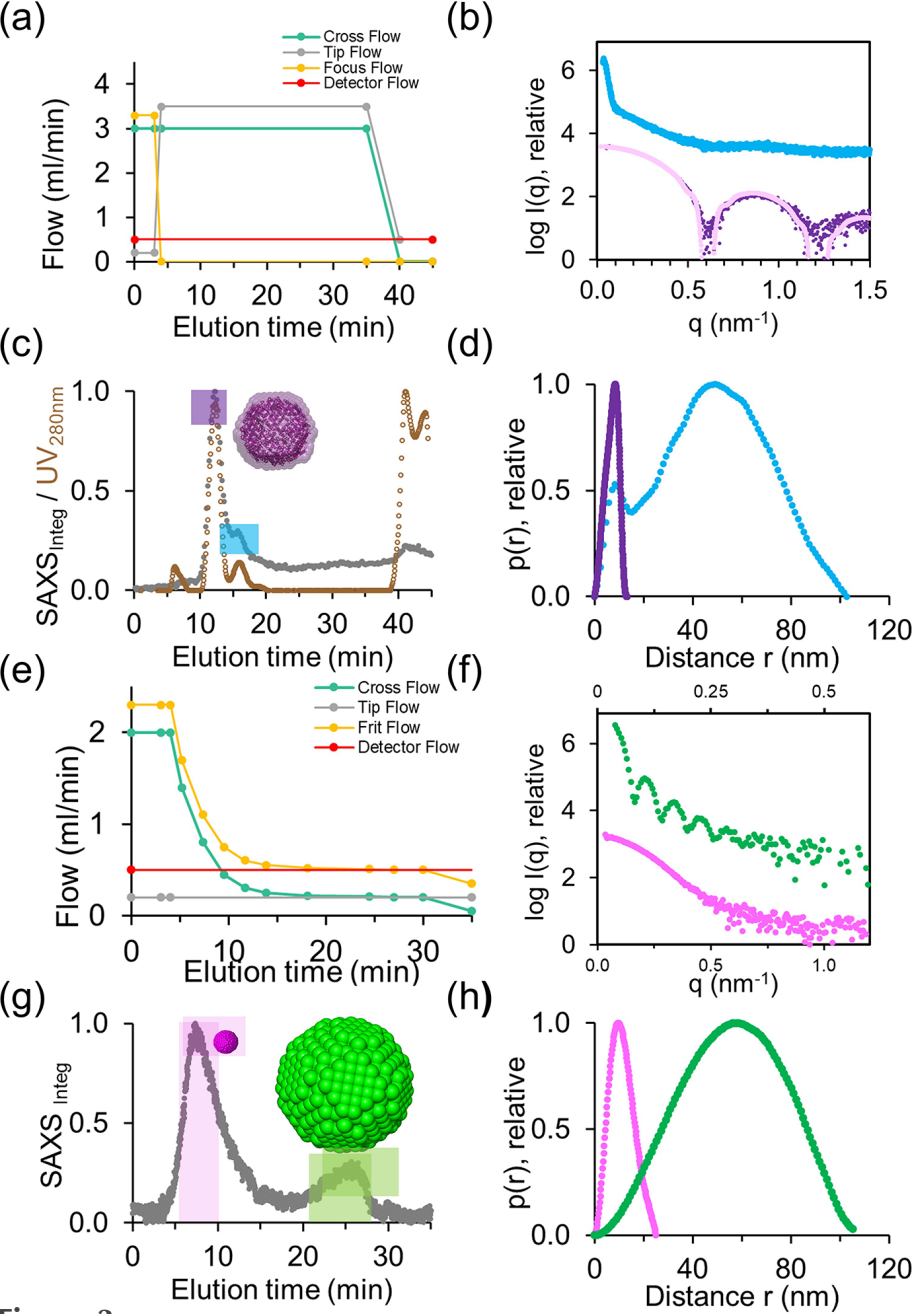
AF4 elution profiles and SAXS fractogram: system validation using model protein apoferritin [(*a*)–(*d*)] and polystyrene (PS) beads [(*e*)–(*h*)]. (*a*) Schematic overview depicting the various flow rate settings during the AF4 separation process of apoferritin. (*b*) Scattering profiles for two elution fractions shown as log*I*(*q*) versus *q*. Profiles are vertically shifted for clarity. The purple curve represents the scattering of the main fraction, which aligns well with the theoretical scattering profile of 24-mer apoferritin (light pink, PDB code 1IER, with *CRYSOL* χ^2^ = 1.8). (*c*) SAXS fractogram displaying the Integrated SAXS intensity (grey, *q*-range for integration 0.25–0.4 nm^−1^) from unsubtracted frames plotted against elution time; overlaid with UV trace (brown). Regions of interest used to generate the scattering curves in (*b*) are highlighted with matching colours. The inset shows an *ab initio* model of the main peak as spheres, overlaid with the atomistic structure of apoferritin (PDB code 1IER). (*d*) *p*(*r*) functions derived from the two SAXS scattering curves in (*b*), demonstrating the size differences between the two highlighted fractions. (*e*) Schematic overview depicting the separation of the PS beads containing a mixture of 20 nm and 100 nm polystyrene beads. (*f*) Scattering profiles obtained from the separated fractions corresponding to the 20 nm beads (magenta) and the 100 nm beads (green). (*g*) SAXS fractogram displaying the integrated SAXS intensity (grey, *q*-range for integration 0.1–0.8 nm^−1^) from unsubtracted frames plotted against elution time. Regions corresponding to the 20 nm and 100 nm beads are highlighted with distinct colours. (*h*) *p*(*r*) functions derived from the SAXS scattering curves for the 20 nm and 100 nm beads, illustrating the differences in size distribution.

For systems smaller than ∼20 nm, a constant Cross Flow rate is maintained throughout the elution phase to achieve effective separation. This approach is demonstrated in Figs. 3 ▸(*a*)–3(*d*), showing the separation of the protein apoferritin. Here, the AF4 method can be divided into three phases, the injection/focusing phase, the elution phase, and the rinse phase. Through these three phases, the Detector Flow rate stays constant at 0.5 ml min^−1^. This is the stream that pushes the particles through the array of detectors and eventually through the SAXS capillary. As mentioned above in formula (1), this is the difference between the Input Flow rate (sum of the Tip Flow and Focus Flow rates) and the Cross Flow rate. During the injection and elution phases, the Cross Flow rate is set constant at 3.0 ml min^−1^ and first turned off during the rinse phase to allow for a more efficient washing out of the system of the particles remaining in the channel.

During the injection phase, 45 µl of sample (apoferritin at 12 mg ml^−1^) was injected with a relatively low Tip Flow rate (0.2 ml min^−1^) while a high Focus Flow rate was applied (3.3 ml min^−1^). This leads to a concentration (focusing) of the sample forming a thin band near the injection site [Fig. S1(*a*)]. Once the Focus Flow rate is turned off after 3 minutes, the Tip Flow rate is then increased to 3.5 ml min^−1^ during the entire elution time and is only reduced to 0.5 ml min^−1^ for the final rinsing step.

The SAXS fractogram and profiles received from the separation of the protein apoferritin are shown in Figs. 3 ▸(*b*) and 3(*c*). The elution profile reveals a prominent peak corresponding to the expected 24-mer cage structure formed by this protein (purple area), along with a shoulder on the right, indicative of the presence of higher-ordered structures (blue area). During the rinse phase, the elution of excess sample confirms that the desired loading amount has been achieved. In contrast to the UV elution profile (brown trace), the baseline of the SAXS fractogram (grey trace) is elevated, potentially indicating capillary build-up due to radiation damage. Nevertheless, the main peak remained unaffected, allowing the collection of high-quality SAXS data suitable for structural characterization through *ab initio* modelling [Fig. 3 ▸(*c*), inset]. The resulting *ab initio* models overlay closely with the atomic structure, and the theoretical scattering curve, calculated from atomistic structures [Protein Data Bank (PDB) code: 1IER], aligns well with the experimental scattering profile derived from the main elution peak [Figs. 3 ▸(*b*) and 3(*c*), purple curve].

The scattering observed in the shoulder (light blue curve) indicates the presence of relatively large particles. The steep slope at very low *q* values suggests that radiation damage has occurred, likely leading to aggregation or structural deterioration of the sample.

This size difference between the scattering from the main peak and the shoulder is further emphasized in the distance probability function, *p*(*r*) [Fig. 3 ▸(*d*)]. The *p*(*r*) function describes the distribution of distances between scattering centres within a sample. It is obtained through an indirect Fourier transform of the scattering data in reciprocal space. The maximum pairwise distance, *D*_max_, represents key structural dimensions, such as the diameter of a sphere or the length of a rod-like object.

Notably, the elution order in AF4–SAXS provides a significant advantage over SEC–SAXS. In SEC–SAXS, larger particles are excluded from the column matrix and elute first. We routinely observe that these particles are often prone to radiation damage, leading to the formation of even larger aggregates. While sensitivity to radiation damage is multi-factorial (Jeffries *et al.*, 2015 ▸; Brooks-Bartlett *et al.*, 2017 ▸), this may be explained by their slower diffusion, leading to longer permanence in the illuminated volume and an effectively enhanced absorbed radiation dose for a constant beam size and exposure time (Hopkins & Thorne, 2016 ▸). These aggregates can adhere to the inner walls of the SAXS capillary, a phenomenon known as capillary fouling (Kirby *et al.*, 2016 ▸), which adds unwanted contributions to the subsequent scattering signal. In contrast, the reverse separation in AF4–SAXS mitigates this issue. Here, the larger and often radiation-damage-prone particles elute only after the data of interest have been successfully collected.

To further compare the separation of apoferritin in AF4 and SEC mode, additional data are presented in Fig. S2. Here, we have adjusted the collection parameters to minimize potential radiation damage by reducing the sample load to approximately half and setting the beam transmission to 60%. Under these conditions, the elution profiles exhibit stable baselines, indicating the absence of residual signal interference or background fluctuations.

The upper panels in Fig. S2 illustrate the effect of varying Cross Flow conditions in AF4–SAXS, while the lower panel shows the results of SEC–SAXS data collection (green trace). The elution profiles are presented on the left, and the corresponding SAXS profiles are displayed on the right. To facilitate a comprehensive evaluation of the different separation approaches, we have overlaid batch-mode SAXS data collected at 0.35 mg ml^−1^. At a Cross Flow of 2 ml min^−1^, the resolution is insufficient to fully separate the main peak from higher oligomeric species, resulting in a poor fit with the theoretical scattering curve (1IER; *CRYSOL* χ^2^ > 1.42). Discrepancies in the low *q*-range indicate that larger components are still present and contribute to the overall scattering profile. At higher Cross Flows, separation improves, yielding a better fit with the theoretical scattering profile (χ^2^ = 1.42). The obtained scattering curve (pink profile) is comparable with the profile obtained with SEC–SAXS [Fig. S2(*h*), green profile]. Since sufficient resolution is achieved with a Cross Flow of 3 ml min^−1^, further increasing the Cross Flow is unnecessary, as it would lead to prolonged analysis times.

The conditions shown here for samples smaller than 20 nm, for which a constant Cross Flow may be applied, are pretty straightforward and have revealed themselves as a good starting point when designing the separation strategy for smaller particles including proteins, polysaccharides, and nucleic acids. Further examples for AF4–SAXS data of proteins collected with this protocol are given in Fig. S3 and are discussed below (Section 2.5).

#### 2.4.2. Decaying Cross Flow

In comparison, for larger particles, the Cross Flow rate is progressively decreased during the elution phase rather than being kept constant. In Figs. 3 ▸(*e*)–3(*h*), we present AF4–SAXS data collected from a mixture of 20 nm and 100 nm polystyrene (PS) beads. In this example, the Cross Flow rate followed an exponential decay, starting at 2 ml min^−1^ and decreasing to 0.2 ml min^−1^ over 30 minutes. This approach is particularly advantageous for separating particles of noticeable different sizes, as it optimizes the separation process by reducing overall run time and ensuring meaningful resolution and separation efficiency (Leeman *et al.*, 2006 ▸). Importantly, the time-dependent Cross Flow rate decrease does not influence the detector baselines neither for the common analytical detectors (MALS, UV, and very sensitive RI detector) nor in the SAXS traces.

An additional feature of using the semi-preparative channel in this example is the different injection mode, referred to as the Frit Inlet mode [Fig. 1 ▸(*b*), inset; Fig. S1(*b*)]. This approach is more sensitive because the sample is injected directly into the flow stream, minimizing contact with the channel walls, especially with the accumulation wall (Moon *et al.*, 1997 ▸; Fuentes *et al.*, 2019 ▸). This replaces the focusing step, reducing potential sample losses, which is particularly beneficial for delicate samples that are prone to agglomeration. The adaptation (omitting the focusing step) is shown in Fig. 3 ▸(*e*). The Frit Inlet channel enables high-resolution fractionation and decreased separation time, but the maximum injectable volume is limited to avoid band broadening.

The fractogram of the SAXS data reveals two peaks with clear baseline separation [Fig. 3 ▸(*g*)]. The first peak corresponds to the 20 nm beads, while the second peak represents the 100 nm beads [Figs. 3 ▸(*f*) and 3(*h*)]. Corresponding SAXS frames were averaged, background subtracted, and used for further analysis (Table 1 ▸).

This separation highlights the influence of the Cross Flow rate on band broadening. Both samples were injected with similar particle weight (0.44% P S20; 1.1% PS 100). The smaller particles, which elute under higher Cross Flow conditions, produce narrower peaks and are therefore more concentrated upon reaching the SAXS capillary. In contrast, the larger particles elute at much lower Cross Flow rates, resulting in broader elution peaks. Consequently, despite their larger size and inherently stronger scattering potential, the SAXS signal of the larger particles is relatively lower compared with that of the smaller particles. In contrast, the UV trace may reach saturation for the large particles.

The separation protocol presented here with exponentially decaying Cross Flow has also proven to be a good starting point when attempting to collect AF4–SAXS data for larger particles (∼20 nm and higher) such as gold nanoparticles, extracellular vesicles, and other particles such as various forms of nano drug carriers.

Besides the influence of varying injection volumes, adaptations to this method are mainly made in terms of tweaking the exponential decay and duration of the Cross Flow. The goal should be to keep the elution time as short as possible to reduce dilution effects, as the injected sample spreads across a broad elution peak (Table 2 ▸). Shorter elution times are also beneficial for the overall use of the beam time; however, it has to be balanced according to the desired resolution. Additionally, the operator should ensure that the overall run time is sufficient so that all sample material is rinsed out of the channel to avoid cross-contamination of the subsequent experiment.

### 2.5. Correlation of MALS and SAXS data for an extended sample characterization

Over the last decade, several beamlines have expanded their SEC–SAXS systems with additional MALS detectors [reviewed by Rosenberg *et al.* (2022 ▸)], enhancing the capabilities for advanced structural analysis. SEC–SAXS–MALS coupling provides a significant advantage by enabling independent characterization of data using information obtained from the MALS detector, either in-line or via a splitter configuration (which requires stable and well defined detector flow rates). The splitter setup is particularly beneficial when using a dRI detector, as it minimizes the inter-detector risk of band broadening caused by the wider capillaries needed to protect the sensitive dRI measuring cell (Graewert *et al.*, 2015 ▸; Graewert *et al.*, 2020 ▸; Graewert & Svergun, 2022 ▸). A similar set-up has been installed at the LiX beamline at NSLS-II (Yang *et al.*, 2020 ▸). Other approaches to supervent band broadening have included the incorporation of the dRI measuring cell after the SAXS capillary as practiced at beamline 13A at the Taiwan Photon Source (Shih *et al.*, 2022 ▸) and at SIBYLS (Richter *et al.*, 2025 ▸).

For our current default AF4 set-up we have omitted the dRI detector and rely only on the UV detector as the concentration detector, allowing the MALS detector to be attached in-line for seamless data acquisition. For samples with low absorption, an independent dRI device is available and can be connected when needed. The inclusion of MALS offers valuable additional benefits, such as providing molecular weight (MW) estimates for proteins and enabling the acquisition of *R*_g_ values through the angular dependency for nanoparticles.

Examples of the addition of UV and MALS signals are shown in Fig. S3. The upper panels depict the elution profiles of two proteins: yeast alcohol de­hydrogenase 1 (ADH) and beta-amylase (β-AM) from sweet potato. Both profiles display a prominent main peak, and the corresponding scattering data align closely with the theoretical scattering curves of the atomistic structures of ADH and β-AM [Figs. S3(*b*) and S3(*d*)].

The SAXS elution profiles align closely with the UV detector signals (absorbance at 280 nm) and MALS signals, as demonstrated here by LS_90°_. This alignment facilitates independent estimation of molecular weight (MW_LS_) from SAXS, offering a reliable means to validate the SAXS data. The intensity of scattered light is directly proportional to the sample concentration and MW. In a calibrated system, the ratio of UV to LS signals can be used to calculate MW. In these cases, both proteins predominantly exist in their tetrameric forms as determined with the MW_MALS_ estimates (Table 1 ▸): 240 kDa for β-AM, and for ADH 144 kDa. Additionally, both samples show a small fraction of monomer subunits. These are detectable with MALS at the start of the elution profile. Further, both samples show a slight shoulder suggesting the presence of higher oligomeric structures.

For larger particles, MALS detectors provide valuable information through angular dependency analysis beyond the typical SAXS *q*-range, enabling *R*_g_ estimation for particles larger than 20 nm. This capability is demonstrated with the elution of 100 nm polystyrene beads [Fig. S3(*e*)]. A total of 50 µl of 1% solution was separated, showing an excellent overlay of SAXS elution profiles with LS_90°_ and UV signals. The UV signal was oversaturated at this concentration and the laser intensity was reduced to 20% to prevent detector saturation, allowing reliable *R*_g_ retrieval. The *R*_g_ values obtained from MALS (brown symbols) agreed well with those derived from SAXS Guinier analysis (green symbols) across the elution peak, showcasing the robustness of this integrated approach.

In summary, the integration of AF4–MALS–SAXS provides complementary insights into molecular weight, size distribution, and sample integrity, enhancing data reliability and interpretation.

### 2.6. Optimization of the AF4 separation

Setting up an AF4 experiment is a complex task due to the many adjustable parameters that influence separation efficiency, sample stability, and data quality. Factors such as Cross Flow rate, channel height, membrane properties, buffer composition, and gradient profiles must be carefully optimized to achieve reliable and reproducible results. The interplay between these parameters makes method development time-consuming, particularly for users unfamiliar with AF4. The Critical Overview published by one of the pioneers of FFF, K.-G. Wahlund, in 2013 (Wahlund, 2013 ▸) highlights both the advantages and limitations of the technique while also providing best practices for method development and data presentation. We highly recommend that experimentalists familiarize themselves with these concepts to better optimize their AF4 separation protocols to suit their specific samples. To assist first-time users, we have summarized selected AF4 optimization steps and key references for getting started (Table 2 ▸). In addition, by familiarizing themselves with BSA separation, experimentalists can develop a more intuitive understanding of how to optimize AF4 parameters for their own samples.

The presence of monomers and dimers in a BSA sollution allows to benchmark the quality of the separation as a function of a few user-modifiable parameters.

**Figure 4:**
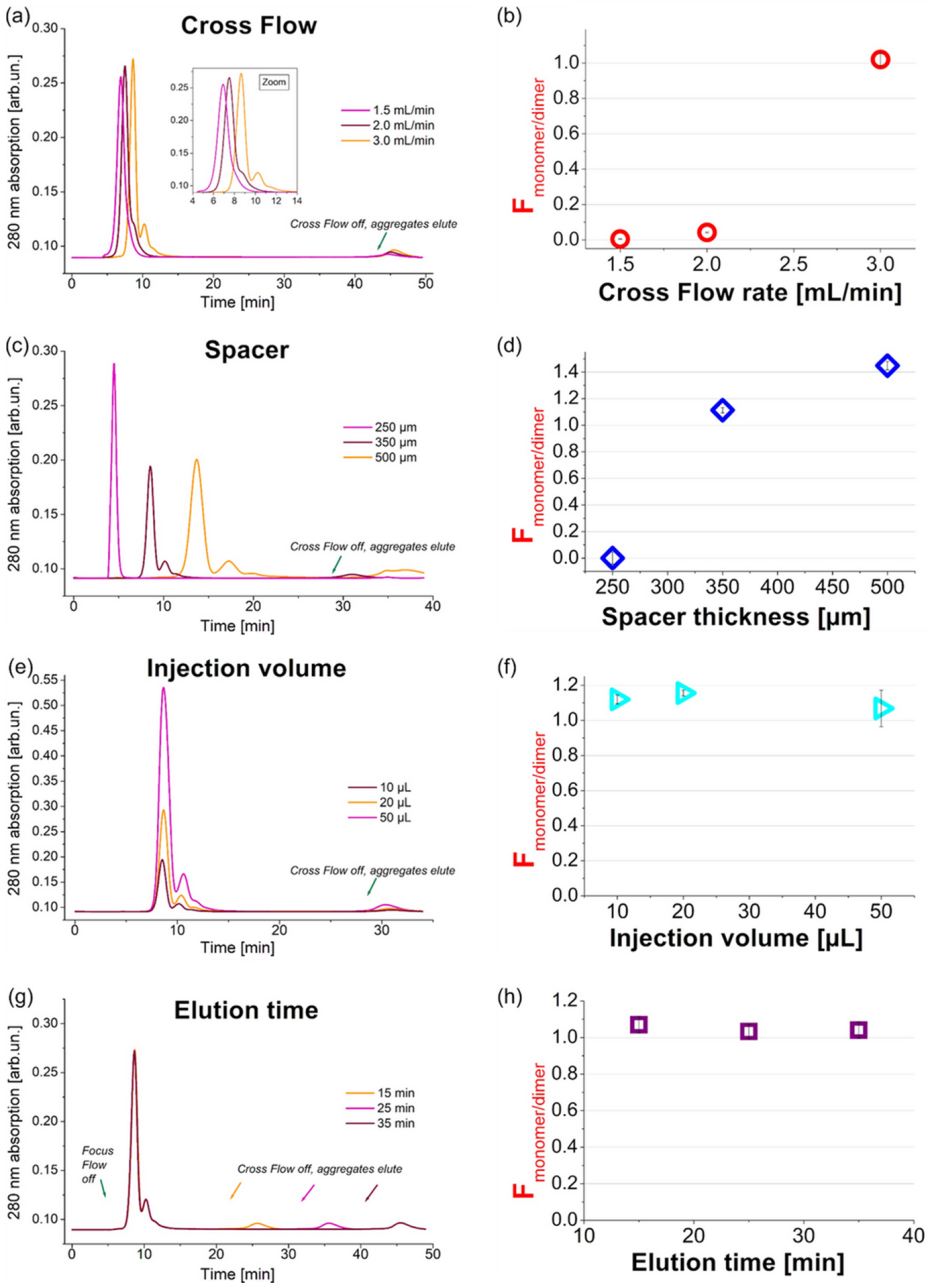
Illustration of the effects of user-modifiable optimization parameters on the separation quality of the benchmarking protein BSA (as monomer, dimer, and higher oligomers). The protein is detected by monitoring UV absorption at 280 nm; a 10 kDa membrane is used for all runs. (*a*) Effect of the rate of the Cross Flow, for 20 µL of BSA 4.6 mg ml^−1^ in PBS buffer, employing a 350 µm-thick channel spacer, and a detector flow of 0.5 ml min^−1^ with a 35 min elution time. Satisfactory separation is reached in this example at 3.0 ml min^−1^ Cross Flow rate. Inset: zoom on the peak region. (*b*) Separation function *F* for the monomer and dimer peaks [after Giddings (1960 ▸)], as a function of the flow rate. (*c*) Effect of the channel height set by the spacer thickness, for 20 µL of BSA 5.0 mg ml^−1^, in PBS buffer, employing for a 25 min elution a Cross Flow of 3.0 ml min^−1^, Detector Flow 0.5 ml min^−1^. (*d*) Separation function *F* as a function of the spacer thickness. (*e*) Effect of the injection volume at constant 5.0 mg ml^−1^ BSA concentration, run in PBS buffer with 350 µm spacer, Cross Flow of 3.0 ml min^−1^, Detector Flow 0.5 ml min^−1^. (*f*) Lack of a significant effect on the separation function *F* within this range of injection volumes. (*g*) Effect of the elution time for a 20 µL injection of 5.0 mg ml^−1^ BSA in PBS, employing a 350 µm spacer, Cross Flow of 3.0 ml min^−1^, Detector Flow 0.5 ml min^−1^. No significant changes in the elution times of the peaks of interest are observed; however, particles large enough not to elute until the Cross Flow is switched off are better separated for longer elution times. (*h*) Lack of significant effect on the separation function *F* for the elution times tested. In all plots showing the values of *F*, the uncertainties are propagated from the fit errors.

The corresponding fractograms are shown in Fig. 4 ▸. To quantify the effects of the parameters on peak resolution, we chose a simple figure of merit for peak separation in analogy with chromatography (Giddings, 1960 ▸; Dvořák *et al.*, 2015 ▸), the separation function *F*, defined as
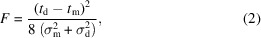
where *t*_m_ and *t*_d_ are the elution times at the centre of the monomer and dimer peaks, respectively, and σ_m_ and σ_d_ are the standard deviations of the monomer and dimer peaks. A good separation is achieved for *F* values around or exceeding 1. The quantities for the calculation of *F* were extracted from the fractograms by fitting the region of the BSA elution peaks with a sum of three Gaussians (for monomer, dimer, and higher oligomers) and a constant background term. For each run parameter tested, *F* values are reported.

Here, we demonstrate the effect of increasing Cross Flow [Figs. 4 ▸(*a*) and 4(*b*)], channel height [Figs. 4 ▸(*c*) and 4(*d*)], injection volume [Figs. 4 ▸(*e*) and 4(*f*)], and elution length [Figs. 4 ▸(*g*) and 4(*h*)].

#### 2.6.1. Cross Flow rate

As already demonstrated above for apoferritin (Fig. S2), it can be appreciated by inspecting Figs. 4 ▸(*a*) and 4(*b*) that the quality of the separation of BSA improves superlinearly increasing the Cross Flow rate. By increasing the Cross Flow from 2 ml min^−1^ to 3 ml min^−1^, the *F* value jumps from ∼0.2 to 1.0, as the dimeric fraction emerges as a well separated peak (orange trace) rather than just a shoulder (pink trace). With higher Cross Flow, the sample experiences stronger retention, leading to longer retention times.

#### 2.6.2. Channel height

Additionally, for the optimal flow rate (here 3.0 ml min^−1^), the separation improves by adjusting the channel height by increasing the spacer thickness [Fig. 4 ▸(*c*)]. An insufficient channel height can result in poor separation even at optimal Cross Flow rate, while an increase in the spacer thickness can further improve the separation, with the side effects of broader and later eluting peaks, which may reduce the throughput due to the necessity of an increased run time in case several measurements are planned. Moreover, peak broadening and delayed elution may be detrimental to the separation quality of biomolecules and particles of larger size. Fig. S4 shows fractograms for model proteins in the range 29–224 kDa employing the three channel heights, highlighting that the specific thickness values to be optimized need to be gauged to the expected molar masses. Additionally, for a constant injected volume and protein concentration the larger spacers cause greater sample dilution (Table S1 of the supporting information). This negatively affects the signal-to-noise ratio of the resulting data when employing SAXS detection.

#### 2.6.3. Sample amount

To increase signal intensity the injection volume can be increased [Fig. 4 ▸(*e*), Table S1]. An increase of a factor two to five in the injected sample volume, within the order of the tens of microlitres, was not found to significantly affect the separation quality at the same BSA concentration, flow and channel height conditions. Minor shifts in the elution time may occur. It must be remembered that exceedingly high sample amounts may cause overloading and sample aggregation. Overloading the AF4 channel can result in peak deformation and decreased resolution. Aggregates (but also pre-existing very large particles) will elute from the channel once the Cross Flow is turned off, and the remaining particles within the channels are rinsed off by a simple tangential detector flow. A slight increase in the aggregate peak, proportional to the increase in sample load, is visible in Fig. 4 ▸(*e*).

Note that when employing the Frit Inlet channel, in which the focusing step is omitted, the injection volume is not freely adjustable [Fig S1(*b*)].

#### 2.6.4. Run time

An increase in elution time, the interval between the end of the Focus Flow and the end of the Cross Flow, delays the rinsing-out of unspecific large particles such as protein aggregates. To minimize the risk that these large scatterers interfere with the peaks of interest, the overall elution time has to be adjusted [Fig. 4 ▸(*g*)]. However, a long elution time is a significant contributor to the overall run time, and it needs to be balanced with beam-time constraints to achieve optimal throughput.

#### 2.6.5. General considerations

These guidelines offer a practical and flexible framework for achieving effective AF4 separation in typical applications of the technique to biological macromolecules. Further selection and optimization guidelines especially addressing the limits and challenges in AF4 method development are summarized in Table 2 ▸.

During the optimization phase, a few control runs may help address potential issues, especially regarding sample–membrane interaction. Such control measurements are shown for AF4–SAXS data collection of apoferritin (Fig. S5). These include running unrestrained elution (*i.e.* Cross Flow set to 0) to assess recovery of the sample after passing the membrane, as well as repetitive measurements after membrane replacement. A clogged membrane can result in incomplete sample elution, leading to decreased resolution, increased baseline noise, or even complete blockage, which would prevent the sample from eluting properly.

## 3. Conclusion

The integration of AF4 with SAXS at the P12 beamline has proven to be an additional advancement for the structural characterization of macromolecules, proteins, nanoparticles, and other nano-scaled complexes. SAXS is an excellent tool for understanding structural properties such as size, shape, and flexibility in solution, but achieving high-quality data depends on the availability of monodisperse samples. This challenge is effectively addressed by the coupling of AF4 to SAXS, which enables efficient separation and SAXS data acquisition, ensuring superior sample quality while maintaining user accessibility.

Building on prior work, such as studies with lipoplex nanoparticles, the AF4–SAXS setup at P12 bridges a critical gap for samples unsuitable for either batch measurements or SEC. The limitations of SEC often stem from issues such as interactions with the stationary phase, dissociation of weakly bound complexes during passage through the column, or incompatibilities with certain buffer components.

While not intended to replace the highly successful SEC–SAXS workflow, the AF4–SAXS platform offers a complementary approach for addressing more challenging systems. AF4 can be more advantageous for systems with (i) broad size distributions (*e.g.* nanoparticles, protein aggregates, and vesicles); (ii) sensitivity to matrix interactions (*e.g.* fragile macromolecules); (iii) high-molecular-weight species prone to clogging SEC columns.

To ease hesitancy in testing this new approach, we have prioritized operational integration with a user-friendly design. Synchronized controls through the *BECQUEREL* interface ensure a seamless and intuitive user experience, aligning with the experimental workflows familiar to the P12 user community. Full automation of processes—including flow regulation, SAXS data acquisition, and sample handling—streamlines operation, reduces complexity, and optimizes throughput. Tools for rapid visualization of results enable users to adapt their data collection strategy in real time, enhancing data quality.

The instrument setup offers significant potential for advancing research in pharmaceutical and life sciences. Intrinsically polydisperse systems such as liposomes, lipid nanoparticles, polymer nanoparticles, emulsions, exosomes, and protein aggregates require accurate, size-resolved characterization for improved understanding and development in pharmaceutical applications. An important challenge is the analysis of micellar systems due to their dynamic equilibrium. Method development should account for concentration-dependent effects and potential structural changes during fractionation, ensuring that separation conditions do not significantly alter micelle properties (Hupfeld *et al.*, 2009 ▸; Glantz *et al.*, 2010 ▸; Vezočnik *et al.*, 2015 ▸). Complementary batch SAXS measurements could help validate micelle integrity and assess the feasibility of AF4 for studying these highly dynamic systems.

First user groups have tested the system on a wide variety of samples, including proteins, nucleic acids, polysaccharides, and larger particles such as nanogold particles, extracellular vesicles, lipoplexes, and lipid nanoparticles, including those used in COVID-19 vaccines. The results from these experiments are currently being prepared for publication. In the latest beam-time call, the option to select the AF4 setup was introduced and has been requested by approximately 10% of users.

Beyond applications to proteins and nanoparticles, the AF4–SAXS setup shows significant promise for environmental studies, where the interactions between macromolecules and particles remain a vital area of investigation. For example, ongoing analyses at P12 focus on mixtures of proteins and nanoparticles, addressing complex samples and particle behaviour in natural environments. A pressing question involves the fate of nanoplastic particles after their uptake into human serum, where they encounter high protein concentrations. These interactions, which can lead to the formation of protein coronas, have implications for particle stability, transport, small-molecules vehiculation, and bioactivity. The versatility of the AF4–SAXS setup makes it a powerful tool for exploring such phenomena, offering critical insights into the environmental and biological fate of nanomaterials.

A current challenge in AF4–SAXS coupling is the sensitivity, particularly for larger particles. At low Cross Flow rates, these particles elute over a longer time frame and thus experience stronger dilution due to the band-broadening effect. Presently, one optimization being explored is the use of an additional device for concentrating the sample [Fig. S1(*c*)]. Both vendors, Wyatt and Postnova, offer such a solution based on the same principle: to concentrate sample constituents after fractionation, Giddings *et al.* proposed the principle of outlet stream splitting in 1983 (Giddings *et al.*, 1983 ▸). Prestel *et al.* published this approach for AF4 in 2006 (Prestel *et al.*, 2006 ▸). The maximum channel height particles progress during fractionation is only within a few micrometres above the accumulation wall. But the nominal channel height is around 350 µm (or 250 µm or 500 µm), which means that most of the liquid amount is particle-free. This particle-free stream is split just before the detector outlet from the channel flow [Fig. S1(*c*)]. It is possible to enhance the detector concentration up to five or six times, and in special cases even higher.

Another option that will be tested in the near future is whether the implementation of a co-flow system (Kirby *et al.*, 2016 ▸) could present an elegant prospect for mitigating the undesired dilution after the sample leaves the MALS outlet capillary and enters the relatively broad SAXS capillary (1 mm).

In conclusion, this robust and accessible setup represents a significant advance in interdisciplinary research, enabling structural insights across fields such as biophysics, materials science, drug development, and environmental science. Future advancements could include further automation of workflows and adaptation for additional sample types, such as complex biofilms or other environmentally relevant systems such as phase-separated condensates. By lowering technical barriers and increasing accessibility, the AF4–SAXS setup at P12 not only enhances structural characterization capabilities but also serves as a vital resource for addressing global challenges, from drug design to nanomaterial safety and environmental impact.

## 4. Materials and methods

### 4.1. Samples

All samples were commercially obtained. From Sigma-Aldrich (Darmstadt, Germany) the proteins were purchased including BSA (cat# 05470) as well as bovine carbonic anhydrase (CA), ADH, sweet potato beta-amylase (bAM) and horse apoferritin (aFER) as part of the Gel Filtration Markers Kit cat# MWGF1000 (Sigma, Darmstadt, Germany). The 20 nm-sized polystyrene beads were purchased from Thermo Scientific (USA, #3020A), the larger 100 nm beads from Sigma-Aldrich (Germany, cat #43302).

The lyophilized protein powders (CA, BSA, bAM, and ADH) were dissolved in preparation buffer PBS. Note that for some measurements 1% glycerol was added to the buffer. Apoferritin is delivered as a solution at ∼50 mg ml^−1^. From this a 1:4 dilution was prepared for further analysis. The final concentrations of all proteins were determined by averaging triplicate UV A280 measurements using the E0.1% values calculated from the amino acid sequence (*ProtParam*; Wilkins *et al.*, 1999 ▸).

The PS beads were suspended in water/detergent solution [0.0125% (*v*/*v*), NovaChem]. The PS 100 nm were either measured directly at 1%, or as a mixture with PS 20 nm beads. In the latter case, the weight percent of PS 100 nm was 1.1% and of PS 20 nm was 0.44%.

### 4.2. AF4–SAXS measurements and analysis

The EMBL P12 beamline at PETRA III (DESY Hamburg, Germany; Blanchet *et al.*, 2015 ▸) is equipped with a POSTNOVA AF2000 AF4 module comprising a UV detector as well as a nine-angle MALS detector (Fig. 1 ▸). A valve system enables seamless switching between HPLC–SAXS mode and conventional batch SAXS measurements using the customized automated sample changer at the beamline [Arinax, France; Round *et al.*, 2015 ▸; Fig. 1 ▸(*b*)]. To minimize band broadening and maintain resolution, the connecting tubes leading to the SAXS capillary were selected with the smallest feasible diameter. For the off-line experiments, the capillary from the MALS detector was simply redirected to the waste bottle.

The beamline’s custom-designed software, *BECQUEREL* (Franke *et al.*, 2012 ▸; Hajizadeh, Franke & Svergun, 2018 ▸), facilitates easy switching between batch and AF4–SAXS modes. It provides remote valve control, integrates the POSTNOVA automatic sample loader, and ensures synchronized communication between the LC system and SAXS data collection.

The injection volumes and concentrations for apoferritin, ADH, aFER, and PS samples are detailed in Table 1 ▸. Standard channels were used for protein AF4–MALS–SAXS runs, while PS bead experiments utilized a frit-inlet semi-preparative channel. In both cases, a regenerated cellulose (RC) membrane with a 10 kDa cutoff was employed (POSTNOVA, Germany). For most cases, a spacer of 350 µm height was used, if not stated otherwise. A detector flow rate of 0.5 ml min^−1^ was maintained to prevent particle accumulation in the SAXS capillary, reducing the risk of radiation-induced buildup.

The control of flow rates for the AF4 separation as well as the analysis of the collected MALS data was performed with the *NovaFFF* software package (POSTNOVA, Germany).

In all instances, UV absorption spectroscopy data were recorded at 280 nm. The MALS system was calibrated to the light scattering of fractionated BSA monomer. The differential RI increment, d*n*/d*c* (ml g^−1^) was set to 0.185 ml g^−1^ for each of the protein samples (Table 1 ▸) and their extinction coefficients were individually calculated from the primary amino acid sequence using *ProtParam*.

An estimate of the dilution of the monomer and dimer peaks compared with the concentrations of the species as injected can be obtained, assuming: (i) no losses of protein mass between measurement of protein concentration in the injected sample and the elution, (ii) constant mass absorption coefficient at 280 nm for BSA monomer, dimers and aggregates along the fractogram, and (iii) no significant change of the monomer and dimer ratios upon dilution. Under these assumptions, a dilution factor *f* can be defined for monomer and dimer in a fractogram following Schure (1999 ▸), as
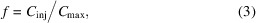
where *C*_inj_ is the concentration of monomer or dimer in the injected sample and *C*_max_ is the concentration of monomer or dimer at peak maximum. For the fractograms in Figs. 4 ▸(*c*) and 4 ▸(*e*), we report estimates for *f* in table form in the supporting information (SI Table 1), employing the fitted Gaussian peaks for monomer and dimer.

The molecular weight estimates, MW_MALS_, were determined from nine-angle MALS scattering intensities combined with the protein concentration determined from UV through the AF4 elution peak of each sample. For the *R*_g_ estimates for MALS that are applicable to larger structures (*R*_g_ > 10 nm) the integrated analysis software from *NovaFFF* was used. Here, the Berry function first-order fit was applied.

For the estimation of the separation, a simple figure of merit for peak separation in analogy with chromatography (Giddings, 1960 ▸; Dvořák *et al.*, 2015 ▸) was applied and is described in more detail above.

For the on-line SAXS measurements, the eluent from the MALS detectors was directed into a 1 mm-diameter quartz capillary housed within the in-vacuum beamline sample exposure unit. For the set of experiments described here, the SAXS data were collected using a Pilatus 6M detector at a sample–detector distance of either 3 m or 6 m and at an X-ray wavelength λ of 0.124 nm. The data were recorded as a sequential set of individual 1 s frames. The total number of frames was determined by the length of the separation run.

For raw data processing, *SASFLOW* and *ATSAS* software (Franke *et al.*, 2012 ▸; Manalastas-Cantos *et al.*, 2021 ▸) were used. Each individual 2D image underwent data reduction (azimuthal averaging) and normalization to the intensity of the transmitted beam to generate 1D scattering profiles plotted as *I*(*q*) versus *q* (where *q* = 4πsinθ/λ and 2θ is the scattering angle).

The *q*-axis was calibrated relative to silver behenate. As a preventative measure, automated washing cycles of the capillary were performed between each AF4–SAXS run to remove the potential and unintended build-up of non-specific debris on the SAXS capillary.

Initial data quality assessment occurs in a similar manner as for the analysis of SEC–SAXS data and is described in greater detail by Graewert *et al.* (2020 ▸). In brief, *CHROMIXS* (Panjkovich & Svergun, 2018 ▸) is launched and either automated or manual inspection is used to determine sample peaks and buffer regions for each run number. The corresponding sample and buffer frames are used for background subtraction, and then scaled and averaged. These ready-for-analysis SAXS profiles can be passed on to the *SASFLOW* pipeline for automated analysis or opened in *PRIMUS* from the *ATSAS* package (Manalastas-Cantos *et al.*, 2021 ▸).

Thus, from each sample region, a set of overall SAXS parameters (potentially including *R*_g_, *D*_max_, MW_SAXS_) can be retrieved as well as the calculation of the real-space distance distribution function [*p*(*r*) profile]. The latter is obtained through an indirect transform of the SAXS profile (Svergun *et al.*, 2013 ▸), and, for a monodisperse dilute macromolecule, assumes values related to the number of lines joining two points at distance *r* within it. Therefore it provides information on its size and shape and can be passed on to the DAMMIF *ab initio* bead modelling routine (Franke & Svergun, 2009 ▸) to obtain a 3D low-resolution reconstruction of the shape. For the protein experiments, the concentration-independent MW_SAXS_ from a combined scattering invariant approach utilizing Bayesian inference was used (Hajizadeh, Franke, Svergun & Jeffries, 2018 ▸).

In addition, the fits of the theoretical scattering curves were used to assess the quality of the obtained SAXS data. For this the program *CRYSOL* (Svergun *et al.*, 1995 ▸) was used as implemented in *ATSAS* 4.0.

In addition, scripts are in place for using *ATSAS* in command line. Experience has shown that it can be beneficial to first average ten frames, then select corresponding buffer frames for subtraction and then running the command AUTORG across all subtracted data allowing the retrieval of the values for the forward scattering *I*(0) and *R*_g_ for each set of averaged frames.

The experimental SAXS data have been deposited with the SASBDB (Valentini *et al.*, 2015 ▸, Kikhney *et al.*, 2020 ▸).

### 5. Related literature

The following reference, not cited in the main body of the paper, has been cited in the supporting information (available online): Witos *et al.* (2020 ▸).

## Supplementary Material

Supporting Figures S1 to S5; Table S1. DOI: 10.1107/S1600577525003959/ju5081sup1.pdf

## Figures and Tables

**Table 1:** Overview of samples and AF4–SAXS parameters

	Proteins			Polystyrene beads	
	Apoferritin	ADH	ß-AM	PS beads 20 nm	PS beads 100 nm
(*a*) Samples					
Organism	Horse	Yeast	Sweet potato	Synthetic	
Buffer	PBS	PBS + 1% glycerol		Water/detergent solution [0.0125% (*v*/*v*), NovaChem]	
Concentration	12 mg ml^−1^	7.7 mg ml^−1^	4.8 mg ml^−1^	0.44%	1.1%
A280 nm extinction coefficient, E0.1%	1.0	1.3	1.77	n.a.	
Expected MW	454 kDa	147 kDa	224 kDa	n.a.	

(*b*) AF4 method					
AF4 channel	Standard			Semi-preparative, Frit-inlet	
Injection	45 µl	50 µl		300 µl (samples injected as mixture)	
Channel height	350 µm				
Membrane	Regenerated cellulose, 10 kDa cut-off				
Cross Flow rates	3 ml min^−1^	4 ml min^−1^		Starting: 2 ml min^−1^	
Crsoss Flow elution profile	Constant			Exponential decay	
Duration	45 min	25 min		35 min	
Detector Flow rate	0.5 ml min^−1^				

(*c*) MALS data collection					
Concentration detector	UV_280_			n.a.	
MW_MALS_	434 kDa	144 kDa	240 kDa	n.a	
*R*_g,MALS_	n.a.			Detectors oversaturated (*R*_g_ confirmed with off-line runs and less load)	

(*d*) SAS data collection					
Source/instrument description	EMBL P12, 6M Pilatus				
Sample temperature (°C)	20°C				
In beam sample cell	1 mm quartz capillary				
Data acquisition/reduction software	*SASFLOW* / *CHROMIXS*				
Detector distance	6 m	3 m		6 m	
Measured *q*-range (*q*_min_–*q*_max_; nm^−1^)	0.01–2.2	0.03–4.4		0.01–2.2	
Exposure time(s),	1 s (∼40 frames averaged)				

(*e*) SAS-derived structural parameters					
	Apoferritin	ADH	ß-AM	PS beads 20 nm	PS beads 100 nm
Methods/software	*ATSAS* (*PRIMUS*)				
Guinier analysis					
*R*_g_ ± σ (nm)	5.4 ± 0.1	3.4 ± 0.1	4.2 ± 0.1	9.4 ± 0.1	48 ± 2
Data point range	12-127	14–99	35–88	1–20	1–6
*PDDF*/*P*(*r*) analysis					
*D*_max_ (nm)	13.3 ± 0.4	9.5 ± 0.4	12.6 ± 0.4	25 ± 3	110 ± 5
Fit assessment	0.89	0.98	0.95	0.67	0.69
MW_SAS_ (kDa)	433 ± 20	110 ± 15	195 ± 20		
Modelling methods					
Software	*CRYSOL*			n.a.	
Atomistic structures	PDB code 1IER	PDB code 4W6Z	PDB code 1FA2	n.a.	
*X*^2^	1.8	1.5	1.5	n.a.	

(*f*) Data and model deposition					
SASBDB IDs	SASDW94	SASDWA4	SASDWB4	SASDWD4	SASDWC4

**Table 2:** Adjustable parameters for AF4 method optimization (A) Default selection. (B) Alternatives available at P12.

Channel (injection strategy)			
Different AF4 channel designs impact separation efficiency and sample recovery. Channels used in commercial AF4 systems are characterized by a trapezoidal shape optimized for flow distribution and enhanced separation resolution (Litzen & Wahlund, 1991 ▸). Different injection strategies are achieved with a focused stream and the Frit Inlet approach (Fig. S1; Moon *et al.*, 1997 ▸; Fuentes *et al.*, 2019 ▸).			
	(A) Standard channel	The most common design is suitable for general nanoparticle and protein separations. It allows a focusing step, enabling the analysis of diluted samples [Fig. S1(*a*)].	
	(B) Preparative channel with Frit Inlet	Reduces sample interaction with the membrane, improving recovery for fragile or adhesive particles [Fig. S1(*b*)].	

Channel height			
Channel height in AF4 is determined by the spacer thickness as well as the swelling behaviour of the membrane affects the channel capacity. Importantly, it also affects the parabolic laminar flow rate profile and thus the separation resolution. With a larger spacer to increase the height a better resolution is obtained but this leads to longer retention times and higher dilution (Wahlund *et al.*, 1986 ▸; Litzén *et al.*, 1993 ▸). The influence of spacer height is visualized in Figs. 4 ▸(*c*) and 4(*d*) and Fig S4.			
	(A) 350 µm	Commonly used for initial experiments to establish baseline conditions and optimize separation parameters. This spacer provides a good balance between resolution, retention time, and sample dilution.	
	(B) 250 µm or 500 µm	A smaller channel height reduces dilution, ensuring a more concentrated sample; increasing the channel height allows for better separation by reducing shear forces and broadening the laminar flow profile. However, this comes at the cost of longer retention times and increased dilution.	

Semi-permeable membrane (ultrafiltration membrane)			
The porous semi-permeable membrane at the accumulation wall, often an ultrafiltration membrane, is a key component in AF4 optimization. The cut-off size (typically 10 kDa) determines which molecules remain in the channel, while the membrane composition influences potential (attractive or repulsive) interactions with the sample. Besides membrane type, the mobile phase may also be optimized to prevent unwanted interactions (Ulrich *et al.*, 2012 ▸; Bendixen *et al.*, 2014 ▸). Control measurements, such as visualizing stuffed membranes, are shown in Fig. S3.			
	Cut-off		
	(A) 10 kDa cut-off	The cut-off is determined by the lowest molar mass of the analytes in the sample with 10 kDa being a good starting point for most applications. For larger analytes the higher cut-off membrane may be more suitable to maintain efficient fractionation (for smaller analytes (<10 kDa), lower cut-offs can be supplied upon request)	
	(B) 50 kDa cut-off		
	Material		
	(A) Regenerated cellulose (RC)	As default, RC membranes are in place. RC has p*K*_a_ ≃ 3.5 and has a negative charge at physiological pH (Schachermeyer *et al.*, 2012 ▸). Depending on the surface charge of the sample, this may need to be adjusted. *e.g.* positively charged particles often require adjustments to the carrier liquid composition or alternative membrane materials (*e.g.* PVDF) to minimize adsorption.	
	(B) Polyethersulfone (PES), olyvinyl­idene difluoride (PVDF)		

Elution profile			
Depending on the size distribution of the particles either a constant Cross Flow or a decaying Cross Flow is used [Figs. 3 ▸(*a*) and 3(*e*)]. The magnitude of both the initial Cross Flow rate (typically between 2 and 5 ml min^−1^) and the profile of the decay determines the optimal elution time point. This is summarized by Wahlund (2013 ▸) and described for experimentally establishing the separation of immunoglobulin G protein by Litzén *et al.* (1993 ▸). The influence of changes in Cross Flow are shown for BSA in Figs. 4 ▸(*a*) and 4(*b*) and for apoferritin in Fig. S2.			
	Constant Cross Flow	For samples smaller than 20 nm, we propose to start with 3 ml min^−1^. Increase the starting flow rate if needed for a better resolution. Adapt the total length of the elution time accordingly [Figs. 4 ▸(*g*) and 4(*h*)].	
	Dynamic Cross Flow	For samples with broad size distributions: a good starting point is Cross Flow of 2 ml min^−1^ and an exponential decay over 25 min.	

Focusing step			
One may start with a default focusing time of 3 minutes. This time can be incrementally decreased until signs of incomplete relaxation are visible (Yang *et al.*, 1977 ▸; Schimpf *et al.*, 2000 ▸). If the sample is unrelaxed, it elutes early, *i.e.* in the vicinity of the void peak.			

Detector Flow			
	(A) 0.5 ml min^−1^	At P12, we propose using 0.5 ml min^−1^ as the default Dector Flow rate to minimize radiation damage in the SAXS capillary, which may occur due to prolonged exposure and sample accumulation.	
	(B) 0.3–0.7 ml min^−1^		

Injection amount			
	For SAXS-coupled AF4 measurements, injecting approximately 300 µg of sample (for both proteins and lipid-based nanoparticles) has proven to be successful. Reported sample amounts range from 150 to 600 µg (Bolinsson *et al.*, 2023 ▸; Graewert *et al.*, 2023 ▸; Börjesdotter *et al.*, 2025 ▸). Overloading the AF4 channel can result in peak deformation and decreased resolution. To prevent this, different injection amounts should be tested, and recovery rates should be determined. Ideally, there should be no shift in the elution time point as injection volume increases (Wahlund, 2013 ▸). Figs. 4 ▸(*e*) and 4(*f*) illustrate the effects of increasing BSA injection volumes. If possible, repeated injections should be performed to assess whether the sample initially adheres to the membrane (Fig. S5).		

Summary			
Adjusting the AF4 separation protocol for challenging systems requires careful consideration of sample recovery, resolution, and SAXS compatibility. While AF4 method development has traditionally been complex, modern FFF software aids in simulation and optimization. However, selecting the optimal carrier liquid and membrane remains experimental. Fortunately, decades of literature provide valuable guidelines, with Wahlund’s (2013 ▸) review offering a strong theoretical and practical foundation.			

## Data Availability

The SAXS data has been submitted to the SASBDB. Complementary data files will be provided upon request. SASBDB entry codes: SASDW94, horse apoferritin from AF4–SAXS measurements; SASDWA4, yeast alcohol de­hydrogenase 1 from AF4–SAXS measurements; SASDWB4, sweet potato beta-amylase from AF4–SAXS measurement; SASDWC4, 100 nm polystyrene beads from AF4–SAXS measurements; SASDWD4, 20 nm polystyrene beads from AF4–SAXS measurements.
