# Supplementary material for: AF4-to-SAXS: expanded characterization of nanoparticles and proteins at the P12 BioSAXS beamline
Source: J Synchrotron Radiat. 2025 Jun 12;32(Pt 4):971–85. doi: 10.1107/S1600577525003959 (PMC12236253; doi:10.1107/S1600577525003959)
Supplement: Supplementary file 1 [file s-32-00971-sup1.pdf]

JOURNAL OF SYNCHROTRON RADIATION

Volume 32 (2025)

Supporting information for article:

AF4-to-SAXS: expanded characterization of nanoparticles and proteins at the P12 BioSAXS beamline

Stefano Da Vela, Kim Bartels, Daniel Franke, Dymtro Soloviov, Tobias Gräwert, Dmitry Molodenskiy, Bastian Kolb, Christoph Wilhelmy, Roland Drexel, Florian Meier, Heinrich Haas, Peter Langguth and Melissa A. Graewert

Supplementary Figure S1

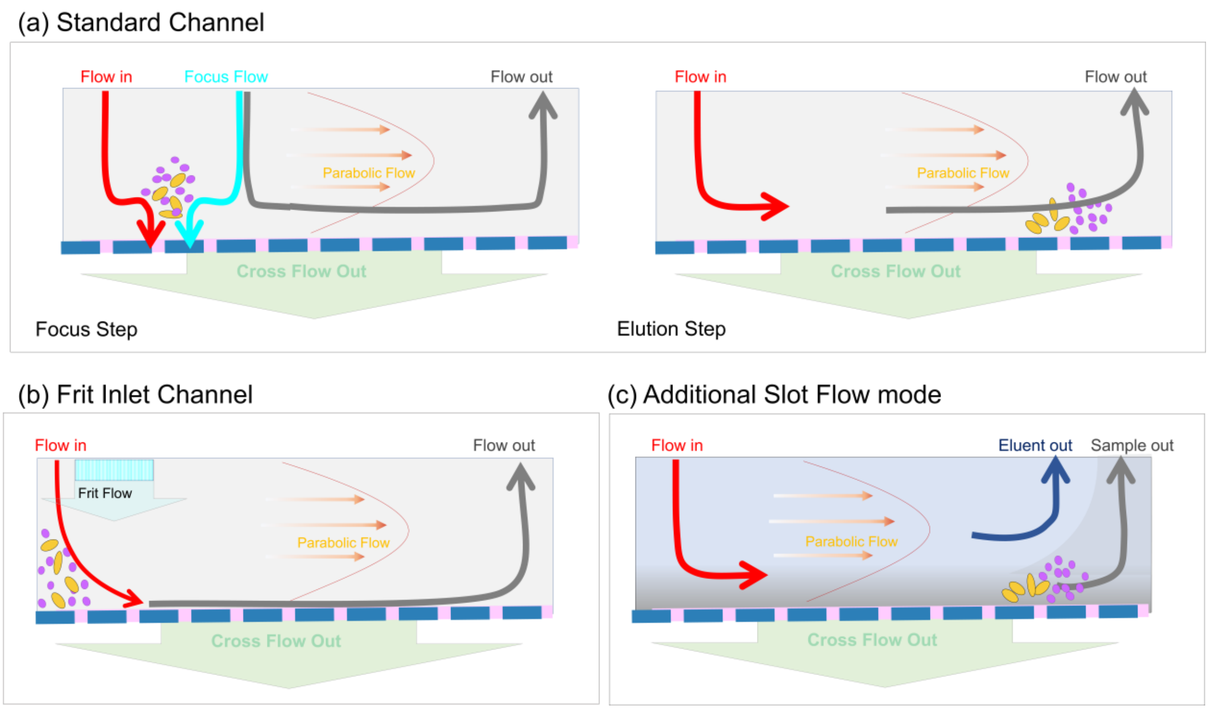

Figure S1 Comparison of Different Flow Strategies in AF4. (a) Standard Channel – During the injection phase and an additional 1–3 minutes, a counter flow (cyan arrow) is applied to concentrate the sample near the accumulation wall. This is referred to as the Focus Flow. Once the Focus Flow is turned off (right panel), the elution step begins, and the sample is carried toward the outlet valve. (b) Frit-Inlet Channel – This approach introduces the carrier liquid through a porous frit (light blue) located at the inlet, ensuring a more uniform and controlled flow across the channel by preventing excessive back-mixing and turbulence. (c) Slot Flow – During fractionation, particles remain within a few micrometers above the accumulation wall, while the nominal channel height is between 250–500 µm. Most of the liquid in the upper part of the channel is particle-free. Just before the detector outlet, this particle-free stream (dark blue arrow) is split from the main flow (gray arrow), enhancing detector concentration up to 5–6 times.

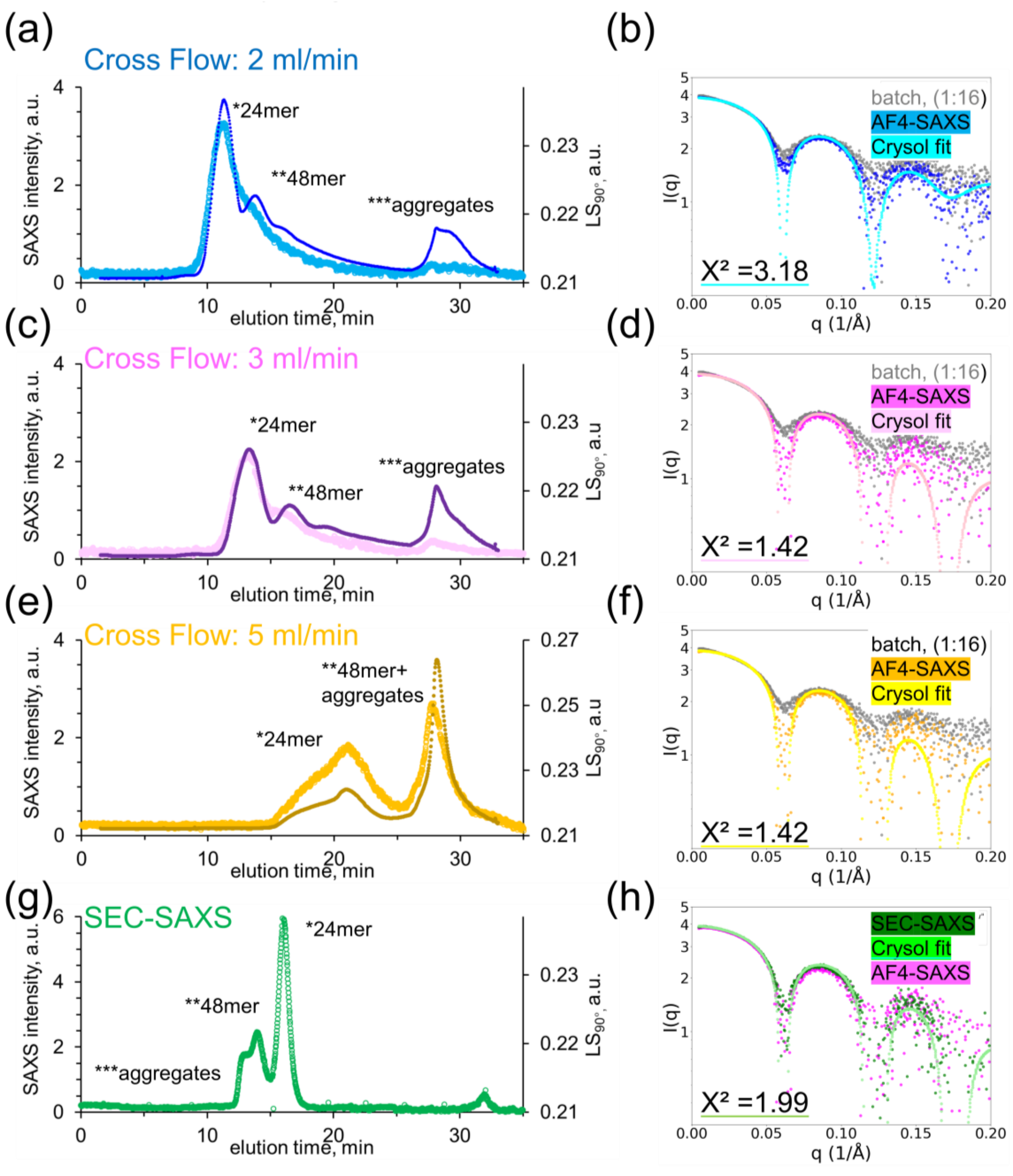

Detector Flow: 0.5 ml/min (AF4); 0.7 ml/min (SEC-SAXS); X-ray transmission: reduced to 60%
Buffer: PBS; Sample: apoferritin, 50 µL at 6 mg/mL in PBS

Figure S2 AF4-SAXS and SEC-SAXS Analysis of Apoferritin – Effect of Cross Flow on Fractionation: 300 µg of apoferritin (50 µL at 12 mg/mL), including a key fraction of higher oligomers, was analyzed using AF4-SAXS and SEC-SAXS. Left panels: SAXS elution profiles with overlaid LS90° traces (solid lines, where available). Right panels: Scattering peaks from the main fraction, overlaid with batch-mode scattering at 0.35 mg/mL (gray). Crysol fits to theoretical scattering curves (1IER.pdb) are shown with corresponding χ^2 values. Effect of Cross Flow on AF4 Fractionation: (a+b) 2 mL/min, (c+d) 3 mL/min, (e+f) 5 mL/min. SEC-SAXS (g+h): The same protein amount was injected onto a Superdex 200 10/300 column. The resulting scattering curve (green) is compared with AF4-SAXS at 3 mL/min Cross Flow (magenta).

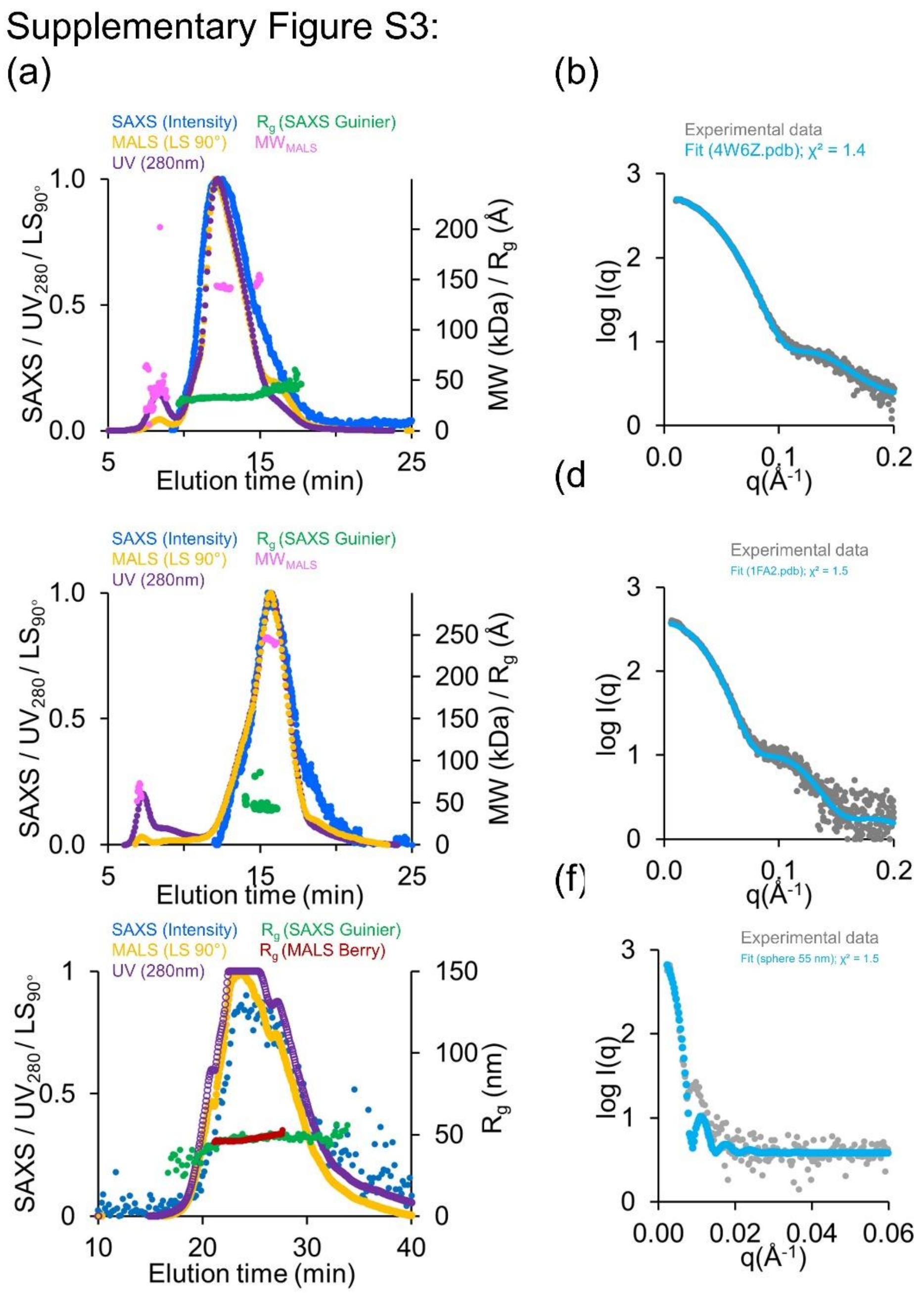

Figure S3 Integration of MALS-derived Information. (a, c, e) SAXS fractograms (blue) and overlay with LS-90° (orange) and UV_{280} (purple) signals for ADH (a), β-AM (c), and 100 nm polystyrene beads (e). For ADH and β-AM, Guinier-derived R_g values from SAXS (green) and MALS-derived molecular weight estimates (MW_{MALS}) are included. For 100 nm PS beads, Guinier-derived R_g values from SAXS (green) align closely with MALS-derived R_g values. (b, d, f) Derived SAXS scattering profiles (grey) and theoretical fits (light blue). Goodness of fit indicated by χ^2 values. For ADH (b), the structure 4W6Z.pdb was used for fitting. For β-AM (d), 1FA2.pdb was used. For PS 100 nm (f), a model of a sphere with a 55 nm radius was applied to fit the experimental scattering profile.

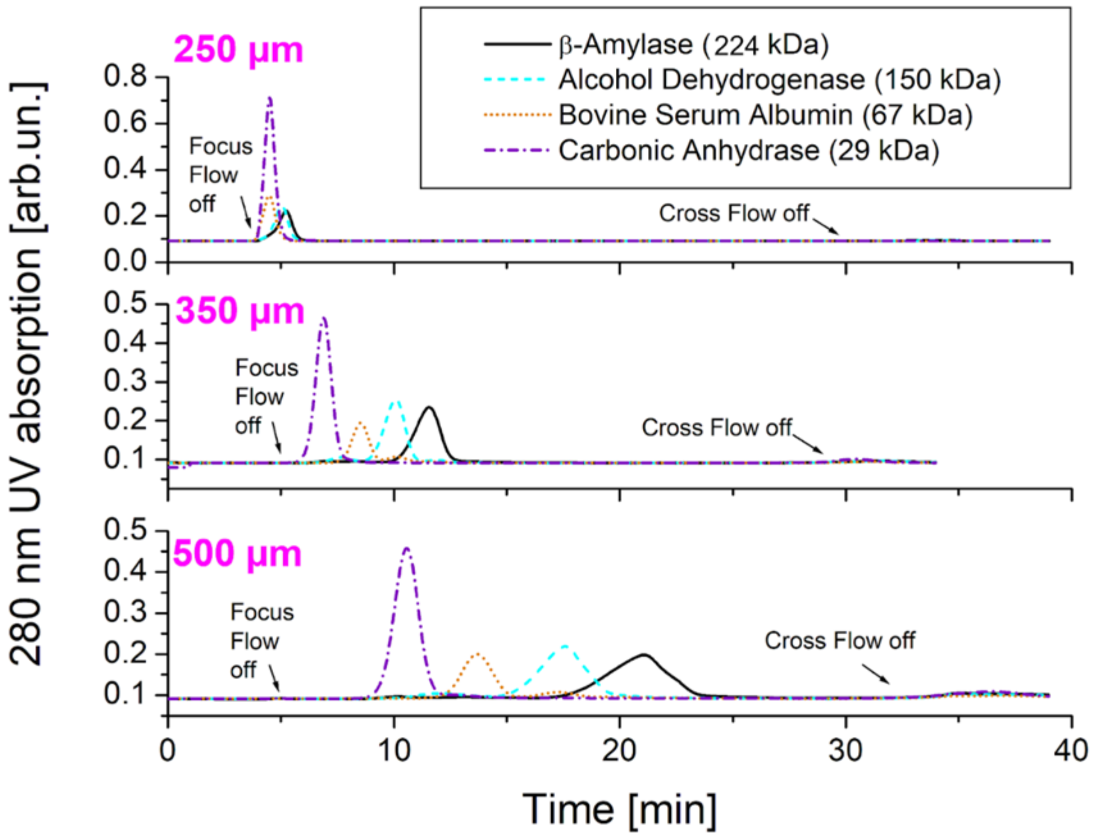

Figure S4 Effect of the channel height set by the spacer thickness for series of proteins ranging in different sizes from 29 - 224 kDa. The protein elution is detected by monitoring UV absorption at 280 nm. A 10 kDa cut-off membrane is used for all runs, Cross Flow rate is set to 3.0 mL/min and the Detector Flow rate to 0.5 mL/min. All runs were set to 45 min. For each sample 3 individual runs were performed at different spacer heights (250 µm, top panel; 350 µm middle panel; 500 µm lower pannel) The samples Protein: BSA (5 mg/mL, 20 uL injection), CA, (5 mg/mL, 20 uL injection), ADH 5 mg/mL, 20 uL injection) and ß-AM (3.2 mg/mL, 20 uL injection).

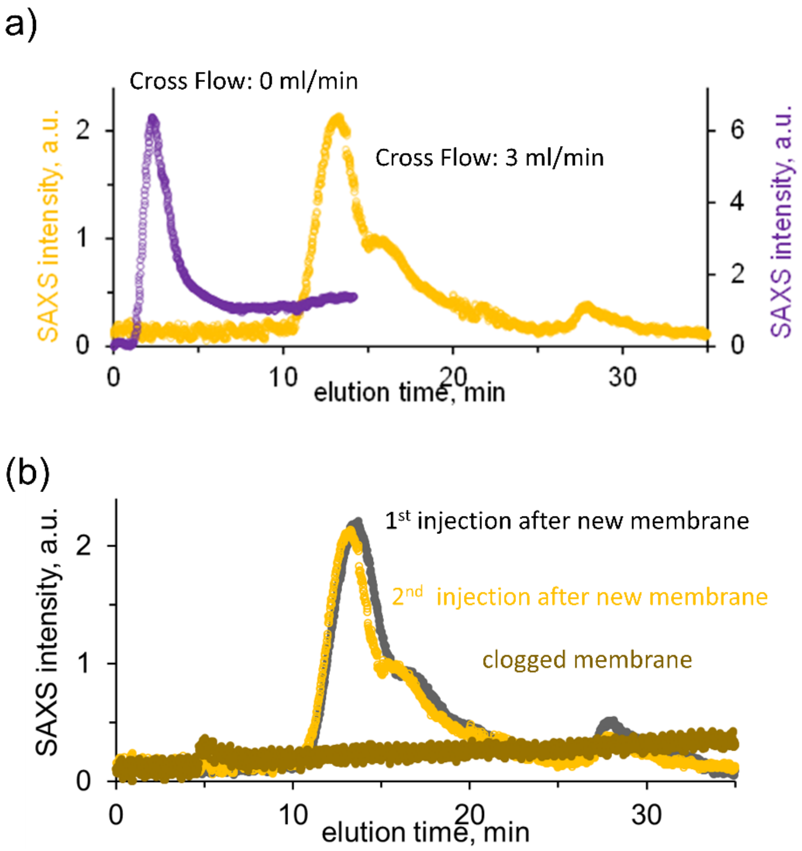

Figure S5 AF4-SAXS Control Measurements. (a) Comparison of AF4-SAXS elution profiles from a non-retained run (Cross Flow off, purple) and a run at 3 mL/min Cross Flow (orange). For clarity, the profiles are shown on separate y-axes, with an overall height reduction by a factor of 3.
(b) Good practice includes repetitive measurements after membrane switching to ensure membrane saturation (grey and orange traces). Lack of sample elution (brown trace) indicates membrane clogging, requiring membrane replacement (Witos *et al. 2020)*.

Table S1 Estimated dilution factors *f*, rounded to whole numbers, as defined in equation **(2)** of the main text, for the fractograms shown in the main text Figs. 4(c) (250, 350, and 500 µm spacer thickness, constant 20 µL injection) and 4(e) (10, 20, and 50 µL injected sample volume, 350 µL spacer thickness).

	Monomer *f*	Dimer *f*
Fig. 4(c), 250 µm spacer	8	9
Fig. 4(c), 350 µm spacer	11	12
Fig. 4(c), 500 µm spacer	21	25
Fig. 4(e), 10 µL injection	23	25
Fig. 4(e), 20 µL injection	12	13
Fig. 4(e), 50 µL injection	6	6
